# Anaerobic solid-state fermentation with *Bacillus subtilis* for digesting free gossypol and improving nutritional quality in cottonseed meal

**DOI:** 10.3389/fnut.2022.1017637

**Published:** 2022-12-08

**Authors:** Jia Li, Tongguo Gao, Zhimin Hao, Xiaojun Guo, Baocheng Zhu

**Affiliations:** ^1^College of Life Sciences, Hebei Agricultural University, Baoding, China; ^2^Feed Microbial Technology Innovation Center of Hebei Province, Baoding, China; ^3^Hebei Bioinformatic Utilization and Technological Innovation Center for Agricultural Microbes, Baoding, China

**Keywords:** cottonseed meal, free gossypol, detoxification, bacterial community structure, anaerobic solid-state fermentation

## Abstract

Microbial fermentation is an effective method to degrade free-gossypol, which is a toxic substance restricting the utilization of cottonseed meal in animal husbandry. However, there are few researches on the nutritional effect and the change of bacterial community on cottonseed meal fermented with anaerobic solid-state fermentation. This study evaluated the effects of fermentation with *Bacillus* sp. on gossypol degradation and nutritional quality improvement in cottonseed meal (CM), as well as the changes of bacterial community structure during fermentation. The strains with high activity for digesting free gossypol were screened from high protease-producing strains preserved in the laboratory. Then the strains which had both the gossypol degradation activity and protease producing activity were selected to degrade macromolecular protein and free gossypol in CM. The unsterilized SSF medium was inoculated with 10^9^ CFU/kg *Bacillus* culture and fermented at room temperature for 14 days. Each group had three parallels. And the effects of anaerobic solid-state fermentation on unsterilized CM was evaluated. Results showed that for the seven strains with high activity for digesting free gossypol and producing protease that were screened, free gossypol content in fermented cottonseed meal (FCM) decreased and acid-soluble protein (ASP) contents increased. Among them, strain M-15 had the best fermentation effect, with the free gossypol degradation rate of 93.46% and acid soluble protein content of 13.26%. M-15 was identified as *Bacillus subtilis*. During fermentation with M-15, the bacterial diversity in CM was reduced, but not significant and the community structure was simpler significantly. The strain M-15 selected in this experiment reduced the free gossypol content and improved the nutritional quality of CM through anaerobic solid-state fermentation, which can be used for industrial large-scale production.

## Introduction

Cottonseed meal (CM) is a by-product of cottonseed oil extraction, with abundant content of crude protein and amino acids. It can be used as a high-quality protein feed resource to alleviate the problem of insufficient protein feed resources in some large cotton-producing countries (1, 2). However, cottonseed meal contains high levels of gossypol. There are two forms of gossypol in cottonseed meal, which are non-toxic combined gossypol and toxic free gossypol. Free gossypol and macromolecular protein, which can decrease digestibility and absorption, thereby constraining the utilization of cottonseed meal in animal husbandry (3–5). Therefore, the detoxification of cottonseed meal and improving the digestion and absorption to extend its application in animal husbandry has always been a topic of concern.

Microbial fermentation is an effective method to degrade free-gossypol. With the microbial fermentation method, we can not only get rid of free-gossypol in cottonseed meal, but also degrade macromolecular protein to improve the nutritional value and palatability of the cottonseed meal (6, 7). So, it is generally agreed that the biological fermentation method is low-cost, effective and safe detoxification method. A lot of species, such as *Saccharomyces cerevisiae, Aspergillus niger, Aspergillus oryzae, Bacillus subtilis*, and *Lactobacillus*, have been applied for removing the gossypol in cottonseed meal (8–10). *S. cerevisiae* and *Lactobacillus* were used to ferment cottonseed meal by Qi et al. (11) and the degradation rate of free gossypol after fermentation reached 77.34%, acid soluble protein content was accounted for 17.61% of crude protein content. Recently, *B. subtilis* has become an ideal strain for cottonseed meal fermentation based on its characteristics and positive fermentation effects (12).

Solid-state fermentation (SSF) is an effective way for *B. subtilis* to degrade free gossypol and macromolecular protein in cottonseed meal (13). Most of the cottonseed meal fermented with SSF adopts aerobic fermentation method using sterilized cottonseed meal, which can shorten the fermentation time, but the incomplete degradation of gossypol will lead to a high residue, and the material loss and cost will be increased. Using unsterilized cottonseed meal for anaerobic solid fermentation can prevent the above restrictions. *Candida utilis* and *Klebsiella oxytoca* were used to ferment cottonseed meal, and the degradation rate of free gossypol was reached to 53.02% after 54 h of aerobic solid fermentation (14). Using *B. subtilis* and *Lactobacillus plantarum*, the degradation rate of free gossypol after 24 h of SSF adopts aerobic fermentation was 52.12% and that increased to 61.58% after 72 h SSF adopts anaerobic fermentation (15). However, there is no research on the nutritional effect and the change of bacterial community on cottonseed meal fermented with anaerobic SSF (16–19).

The degradation of gossypol and the improvement of nutritional properties in cottonseed meal depend on the gossypol degradation activity and protease activity of microorganisms. Therefore, the *Bacillus* sp. strains selected for cottonseed meal fermentation must have both the gossypol degradation activity and protease producing activity. Our laboratory is the “Innovation Technology Center of Feed Microorganisms in Hebei Province, China,” which is the key laboratories in Hebei Province, China. In previous study, a number of *Bacillus* strains with protease producing activity screened from soil in an oil mill, for the production of fermented protein feed. In this paper, a strain with a relatively higher activity for digesting free gossypol was selected from the high protease producing strains previously preserved in our laboratory. Then the strain was used to ferment unsterilized cottonseed meal using anaerobic SSF. And the changes in content of free-gossypol, nutritional quality and bacterial community structure of FCM was investigated.

## Materials and methods

### Chemicals, reagents, and strains

Cottonseed meal was purchased from Manas Yintian Cotton Industry Co., Ltd. (Manas County, Changji Hui Autonomous Prefecture, Xinjiang, China).

Liquid medium with free gossypol: Free gossypol 5.0 g, Beef extract 1.0 g, Peptone 2.0 g, (NH_4_)_2_SO_4_ 2.0 g, MgSO_4_⋅7H_2_O 0.2 g, NaH_2_PO_4_⋅H_2_O 0.5 g, CaCl_2_⋅2H_2_O 0.1 g, K_2_HPO_4_ 0.5 g, H_2_O 1000 mL, pH 7.2–7.4.

Aniline, isopropanol, n-hexane, 3-amino-1-propanol, glacial acetic acid and anhydrous ethanol were purchased from Sigma-Aldrich (St. Louis, MO, United States). All the chemicals were of analytical grade locally.

*Bacillus* sp. strains named: M-1, M-2, M-3, M-4, M-5, M-6, M-7, M-8, M-9, M-10, M-11, M-12, M-13, M-14, M-15, M-20, M-22, M-28, M-35, M-40, M-44, H-1, H-2, H-3, H-4, S-1, S-2, S-3, Z-26, Z-27, A1, A2, A3, B1 and B2 were screened as protease secreting strains and stored in our laboratory. All strains were microscopically examined using Gram- and Schaeffer–Fulton stains to confirm that they were *Bacillus* spp.

### Screening of gossypol degradation strains

For primary screening, the strains were inoculated on the plate with about 20 mL solid medium with free gossypol as the sole carbon source and cultured at 30°C. The strains which could grow in the plates were inoculated in 100 mL liquid medium with 0.5 g free gossypol and oscillated at 37°C, 180 r/min for 72 h for second screening. Contents of free-gossypol in the liquid medium before and after fermentation were determined and recorded by the aniline method (20, 21). The free-gossypol in the cottonseed meal was extracted by mixed solution of isopropyl alcohol and n-hexane according to Aniline method, and the content of free-gossypol in cottonseed meal was 980 mg/kg. According to the determination results, the strains with a relatively higher activity for digesting free gossypol were taken and stored.

### Effect of cottonseed meal solid-state fermentation

The unsterilized SSF medium [cottonseed meal 80.0%, corn meal 17.0%, (NH_4_)_2_SO_4_ 3.0%] was inoculated with 10^9^ CFU/kg *Bacillus* culture, mixed with an equal volume of water, then put into buckets (6 kg), compacted and sealed, and fermented at room temperature for 14 days. Each group had three parallels.

### Chemical composition of the fermented cottonseed meal

Samples before and after fermentation were taken, contents of crude protein, acid-soluble protein, free-gossypol, neutral detergent fiber (NDF), acid detergent fiber (ADF), ASH, and the pH of the samples were determined. The contents of free-gossypol were determined by the aniline method (20). The crude protein contents were determined by the Kjeldahl method (22). The acid-soluble protein (ASP) contents and the pH of the samples were determined using the methods (23) described by Xuan et al. Neutral detergent fiber (NDF) contents, acid detergent fiber (ADF) contents, and ash contents of the samples were determined according to the methods (24) described by Ma et al.

### Identification of the strain

DNA of the strain was extracted with the CTAB method (25). Then the 16S ribosomal RNA fragment of the strain was amplified using forward primer (5±-AGAGTTTGATCCTGGCTCAG-3±) and reverse primer (5±-CTACGGCTACCTTGTTACGA-3±). The PCR system and condition was performed according to reference (26). The PCR products were sequenced by Huada Gene Technology Co., Ltd. And the sequence alignment and analysis were carried out with the tool (BLAST) services provided by the National Center for Biotechnology Information (NCBI)^1^ (27, 28).

### High-throughput sequencing of 16S ribosomal RNA gene

According to the result of acid-soluble protein (ASP) and free-gossypol, samples of 14-day fermentation (FCM) as best fermentation group and control group (CK) were sent to compare the bacterial communities by high-throughput sequencing of 16S ribosomal RNA (rRNA) genes.

Genomic DNA was extracted using the rapid bacterial genomic DNA isolation Kit (Shanghai Sangong). The 16S rRNA V3 + V4 region was amplified with the following primers: forward primer 341F: 5±-CCTAYGGGRBGCASCAG-3± and reverse primer 806R: 5±-GGACTACHVGGGTWTCTAAT-3±. The PCR condition included initial denaturation at 95°C for 3 min; 27 cycles of 95°C for 30 s, 55°C for 30 s, and 72°C for 45 s; and final extension at 72°C for 10 min (29, 30). The PCR products were sequenced by Shanghai Meiji Biomedical Technology Co., Ltd. (Shanghai, China) using Illumina HiSeq 2500.

### Sequence analysis

The sequences with high quality were clustered into different operational taxonomic units (OTUs) based on a 97% sequence similarity using the Uparse software (version 7.0.1090).^2^

And the Ribosomal Database Project (RDP) classifier and Silva (release 138)^3^ reference gene database were used for species annotation analysis, and the community species composition of each sample under each taxonomic level was counted.

Alpha diversity (Goods-coverage, Chao1, Ace, Shannon, Simpson diversity indices) was calculated using Qiime software (Version 1.9.1).^4^ The dilution curve and Shannon curve was drawn with R software.

The beta diversity distance matrix was calculated using Qiime software (Version 1.9.1). Principal coordinate analysis (PCoA) diagram was drawn with R software (version 3.3.1).

### Statistical analysis

All experiments were performed in triplicate. The data were expressed as mean ± SD. Statistical analysis was done using oneway ANOVA and Duncan’s test at a value of 0.05 using SPSS (SPSS Inc., Chicago, IL, USA).

## Results

### Strain screening

**FIGURE 1:**
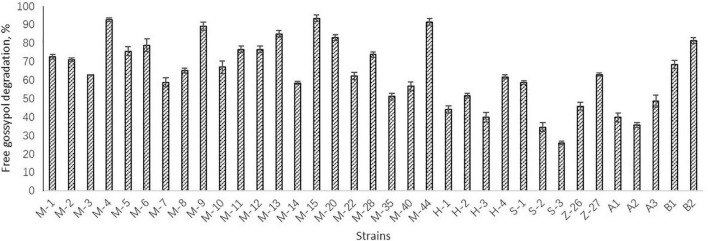
Degradation activity of free gossypol of strains. Values are expressed as averages ± standard deviation (SD).

Preliminary screening resulted in 15 *Bacillus* strains which could grow in the solid medium with free gossypol as the sole carbon source. The degradation activity of free gossypol of the 15 strains were tested, with results shown in Figure 1. The strains M-4, M-9, M-13, M-15, M-20, M-44, and B2 with high degradation activity of free gossypol were chosen for cottonseed meal fermentation, whose degradation rate of free gossypol was higher than 80% (Figure 1).

### Changes of the contents of free gossypol and acid-soluble protein after fermentation

**FIGURE 2:**
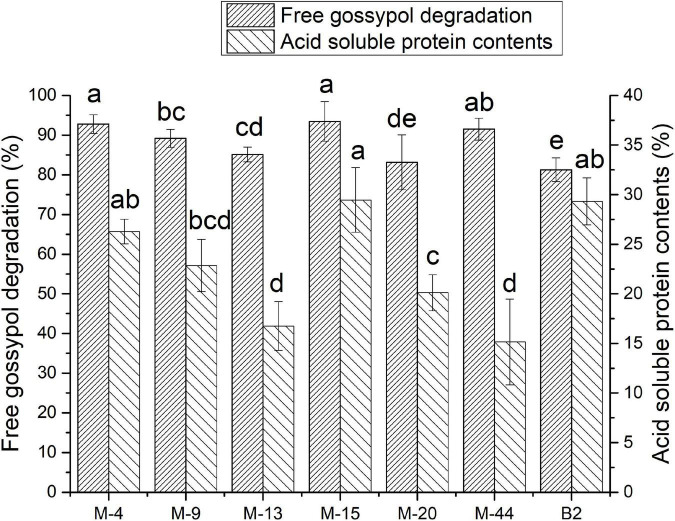
Free gossypol degradation rate and acid-soluble protein (ASP) contents of cottonseed meal (CM) and CM fermented with M-15 (FCM). Values are expressed as averages ± standard deviation. Different letters indicate significant differences (*P* < 0.05) among groups.

The free gossypol degradation and ASP in cottonseed meal fermented by seven strains were shown in Figure 2. The free gossypol content in FCM reduced to 51.26–146.98 mg/kg after fermentation. The FCM fermented with M-4, M-15, and M-44 had a less free gossypol remain at 56.37, 51.26, and 66.58 mg/kg after fermentation, decreased by 92.81, 93.46, and 91.51%, respectively, compared with CK.

Acid-soluble protein contents of FCM reached 8–15% after fermentation. FCM fermented with M-4, M-15, and B2 had a higher ASP content at 11.88, 13.26, and 13.52%, which was accounted for 26.28, 29.45, and 29.33% of crude protein content. Respectively, ASP content in CK was accounted for 5.8% of crude protein content.

### Results of strain identification

**FIGURE 3:**
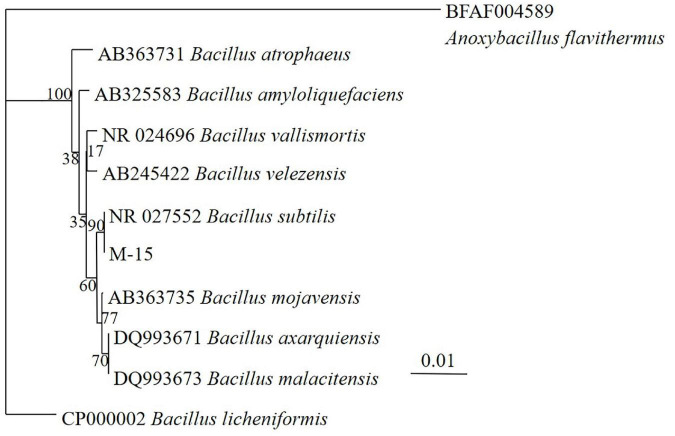
Neighbor-joining tree based on 16S rDNA sequences showing relationship between the tested bacterium strain M-15 and related strains.

After fermentation, M-15 demonstrated improvements in free gossypol degradation and ASP content increase. Based on 16S rRNA gene sequences (Table 1 and Figure 3), combined with morphological and biochemical characteristics, M-15 was identified as *B. subtilis*. The 16S rRNA gene sequence was submitted to the GenBank database (accession number: HQ401271).

**TABLE 1:** Similarity of the sequences of 16S rDNA between strain M-15 and reference strains.

Sequence number	Species name	Strain number	Similarity (%)
BFAF004589	*Anoxybacillus flavithermus*	DSM 2641	89.68
AB325583	*Bacillus amyloliquefaciens*	NBRC 15535	99.48
AB363731	*Bacillus atrophaeus*	NBRC 15539	99.17
DQ993671	*Bacillus axarquiensis*	LMG 22476	99.78
CP000002	*Bacillus licheniformis*	ATCC 14580	97.73
DQ993673	*Bacillus malacitensis*	LMG 22477	99.78
AB363735	*Bacillus mojavensis*	NBRC 15718	99.78
NR_027552	*Bacillus subtilis*	DSM 10	100.00
NR_024696	*Bacillus vallismortis*	DSM 11031	99.70
AB245422	*Bacillus velezensis*	LMG22478	99.85

### Relevant indexes of fermented cottonseed meal

Our results showed that it only takes 14 days to degrade free gossypol to 51.26 mg/kg in cottonseed meal fermented with strain M-15. The crude protein content of the cottonseed meal after fermentation reached levels of higher than 45.03%.

Content of crude fiber, neutral detergent fiber (NDF), acid detergent fiber (ADF) and ash in FCM decreased significantly by 10.84, 14.33, 18.32, and 8.90% compared with that in CK. The pH value in FCM group was 4.68 and that in CK group was 6.55 (Table 2).

**TABLE 2:** Relevant indexes of fermented cottonseed meal.

Item	CK	FCM
Free gossypol content (mg/kg)	784 ± 0.74^a^	51.26 ± 0.88^b^
Rate of free gossypol degradation (%)	−	93.46 ± 0.11
Crude protein content (%)	−	45.03 ± 0.72
ASP (%)	5.8 ± 0.03^a^	13.26 ± 0.51^b^
Crude fiber (%)	16.97 ± 0.40a	15.13 ± 0.20^b^
NDF (%)	14.17 ± 0.84^a^	12.14 ± 0.65^b^
ADF (%)	21.13 ± 0.90^a^	17.26 ± 0.40^b^
Ash (%)	7.91 ± 0.18^a^	7.20 ± 0.03^b^
pH value	6.55 ± 0.03^a^	4.68 ± 0.02^b^

CK: cottonseed meal (CM); FCM: cottonseed meal fermented with M-15 (FCM). Values are expressed as averages ± standard deviation. Different letters indicate significant differences (*P* < 0.05) among groups.

### Changes in flora after fermentation with M-15

Samples of cottonseed meal (CM) and cottonseed meal fermented by M-15 (FCM) were sent to the sequencing company to detect the change of bacterial structure during fermentation.

#### Sequencing data and cluster analysis

**FIGURE 4:**
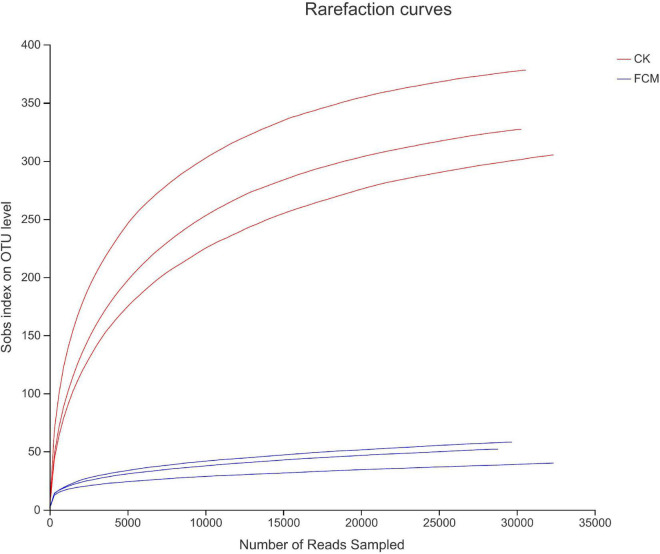
Rarefaction curves based on OUT level. Rarefaction curves based on observed species value. The rarefaction curve was plotted where the X-axis represents the number of clones (sequences) and the Y-axis represents the number of OUT [CK: cottonseed meal (CM); fermented cottonseed meal (FCM): FCM].

**FIGURE 5:**
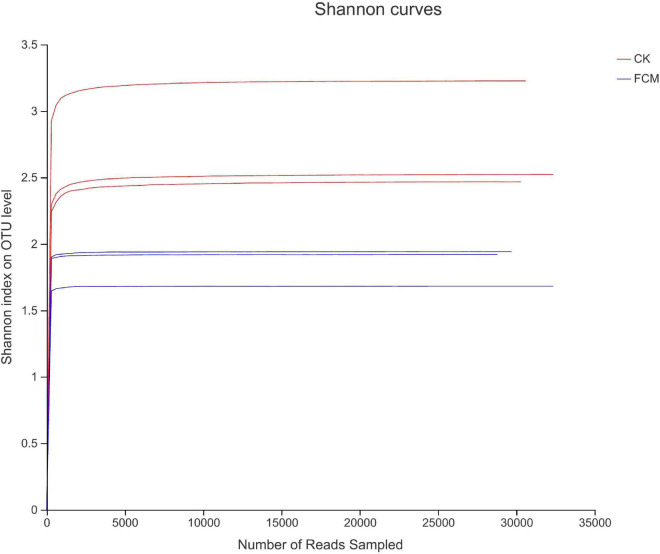
Shannon curves based on OUT level [CK: cottonseed meal (CM); fermented cottonseed meal (FCM): FCM].

Figure 4 shows the rarefaction curves of CK and FCM samples. Figure 5 shows the Shannon curves of CK and FCM samples. With the continuous deepening of sequencing, it can be seen that curves gradually plateaued, becoming parallel to the X-axis. It indicates that the depth of sequencing had included all OTU in the sample. Table 3 shows that coverage was more than 99%, combined with Shannon curves, which indicates that the amount of sequencing data is large enough to reflect the vast majority of bacterial diversity information, and the sequencing meets the analysis requirements.

**TABLE 3:** Samples alpha diversity analysis index.

Sample\Estimators	Shannon	Simpson	Ace	Chao1	Coverage
CK_1	2.467083	0.281057	355.9936	358.5714	0.998272
CK_2	2.521669	0.200116	341.5988	329.1754	0.998356
CK_3	3.226665	0.150627	404.4599	406	0.998388
FCM_1	1.919611	0.199272	69.70483	67	0.999475
FCM_2	1.941903	0.182492	76.48197	73.11111	0.999422
FCM_3	1.68169	0.257192	79.10488	59.5	0.999598

CK: cottonseed meal (CM); FCM: cottonseed meal fermented with M-15 (FCM).

#### Bacterial alpha diversity analysis

Alpha diversity is used to analyze the diversity, richness and coverage of species in the microbial community, including Shannon, Simpson, ace, chao1 and coverage. Among them, ace and chao1 reflects population richness, Shannon and Simpson reflect population diversity (30, 31). The alpha diversity indices of the samples are shown in Table 3. The results of the alpha diversity *t*-test (*P* < 0.05) in the FCM group and CK group are shown in Table 4. The Ace index and Chao index were significantly lower in the FCM group than those in CK group. The Shannon index were lower, and the Simpson index was higher in the FCM group than those in the CK group, but not significant. It indicates that the bacteria in the CK group had a higher abundance and diversity. But the fermentation with strain M-15 did not significantly change the diversity of cottonseed meal bacteria, only significantly change the abundance of cottonseed meal bacteria. The results showed that the bacterial diversity in cottonseed meal was reduced, but not significant and the community structure was simpler after fermentation.

**TABLE 4:** Differences in samples alpha diversity.

Sample\Estimators	Shannon	Simpson	Ace	Chao1
CK	2.739	0.211	367.351	364.582
FCM	1.848	0.213	75.097	66.537
*P*-value	0.113	0.970	0.004	0.008

CK: cottonseed meal (CM); FCM: cottonseed meal fermented with M-15 (FCM).

#### Operational taxonomic unit-specific analysis

**FIGURE 6:**
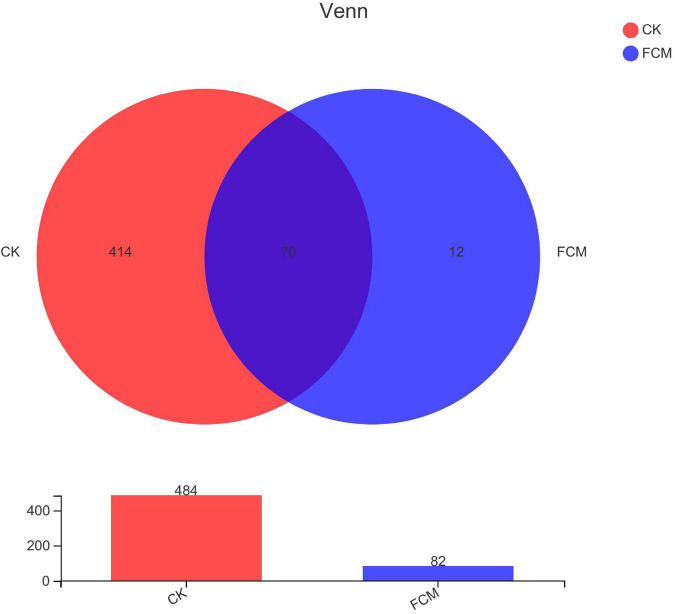
Venn diagram analysis based on operational taxonomic units (OTUs) for different groups. The number of overlapping parts is the number of OTUs shared between the two samples [CK: cottonseed meal (CM); fermented cottonseed meal (FCM): FCM].

The statistical analysis of biological information was performed with OTU at 97% similarity level (Figure 6). There were 496 OTUs in the two groups, 484 OTUs in the CK groups, 82 OTUs in the FCM groups. The groups were compared in pairs. There were 414 OTUs of specific bacteria in the CK group, accounting for 83.47%, 12 OTUs of specific bacteria in the FCM group, accounting for 2.42% and 70 OTUs of the same bacteria in the two groups, accounting for 14.11%. After fermentation, the diversity of bacterial community in cottonseed meal decreased significantly, and the composition of bacterial community in FCM group was significantly different from that in CK group.

#### Analysis of community structure at the gate level

**FIGURE 7:**
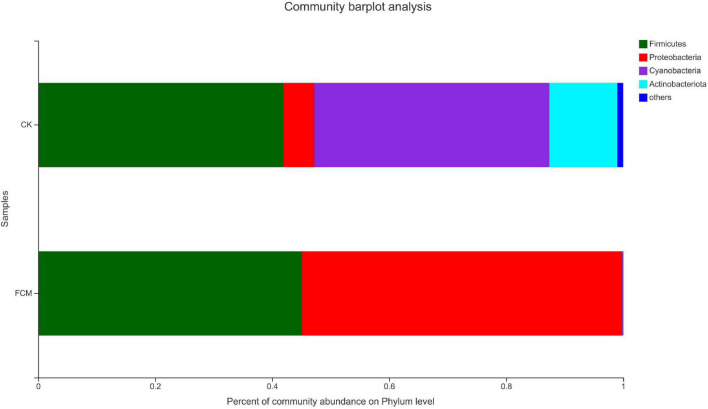
Column diagram of bacterial community at the phylum level. The abundance is represented in terms of the percentage of the total effective bacterial sequences in the sample [CK: cottonseed meal (CM); fermented cottonseed meal (FCM): FCM].

Figure 7 is the column diagram of bacterial community in each group of samples at the gate level, which is used to reflect the dominant species and the relative abundance of each dominant species. It is observed from Figure 7 that the samples mainly contain 4 dominant phyla, including Firmicutes, Proteobacteria, Cyanobacteria and Actinobacteria. In the CK group, Firmicutes (41.95%) and Cyanobacteria (40.18%) were the dominant phyla, while Proteobacteria (5.23%) and Actinomycetes (11.63%) accounted for relatively less. In the FCM group, the proportion of Cyanobacteria (0.11%) and Actinomycetes (0.03%)in the fermented cottonseed meal decreased significantly, Firmicutes (45.11%) increased slightly, Proteobacteria (54.75%) increased significantly, becoming the dominant phyla.

#### Analysis of community structure at the genus level

**FIGURE 8:**
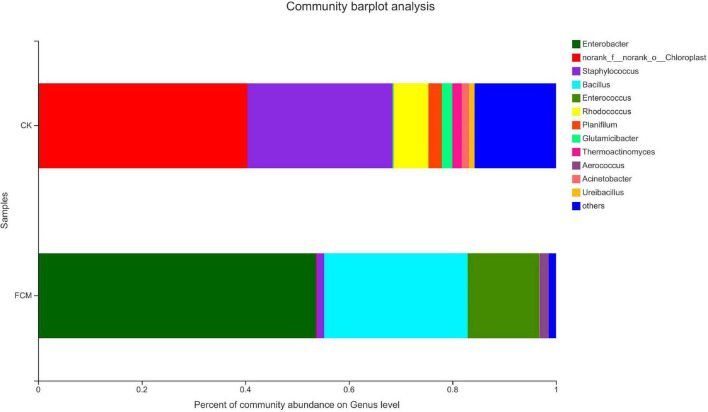
Column diagram of bacterial community at genus level. The abundance is represented in terms of the percentage of the total effective bacterial sequences in the sample [CK: cottonseed meal (CM); fermented cottonseed meal (FCM): FCM].

Figure 8 shows the column diagram of bacterial community in the samples at the genus level. In the CK group, norank_f_norank_o_ Chloroplast (40.18%) was the dominant genus, which was a genus without specific name, and named by the Majorpio cloud platform. In the FCM group, the abundance of Enterobacter increased to 53.64%, becoming the dominant genus. The abundance of *Bacillus* also increased significantly, reaching 27.73%.

#### Beta diversity analysis

**FIGURE 9:**
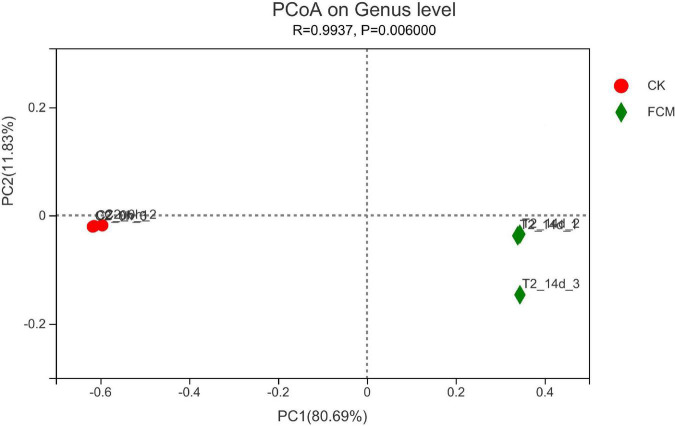
Principal coordinate analysis (PCoA) analysis at genus level. In each sample name, CK and fermented cottonseed meal (FCM) represent the sample from CM and FCM, respectively. Points with the same pattern represent the three replicate samples from the same group.

Figure 9 is the PcoA diagram at genus level, which can be used to study the similarity and difference of community composition in samples through principal coordinate analysis. The results in figure 9 show that two principal components are screened, with PC1 variance contribution rate of 80.69% and PC2 variance contribution rate of 11.83%. The difference between groups is greater than the difference within groups, indicating that the grouping is meaningful. FCM group is more scattered than CK group, and the flora changes greatly, indicating that the bacterial community changes significantly after fermentation compared with that before fermentation.

## Discussion

### Effect of different species on the quality of fermented cottonseed meal

A lot of species has been applied for removing the gossypol in cottonseed meal. Hong et al. (32) found that *S. cerevisiae, B. subtilis*, and *Lactobacillus* are the three most effective types of microorganisms for detoxification of cottonseed meal, and different strains have different effects on the quality of fermented cottonseed meal. The cottonseed meal fermented by *Lactobacillus* has acid flavor. During the fermentation process, a large amount of lactic acid, acetic acid and other organic acids will be produced, which reduce the pH in FCM, improve the palatability in FCM, inhibits the growth of harmful bacteria, and prevents FCM from corruption and mildew.

The cottonseed meal fermented by *S. cerevisiae* has acid flavor or alcohol flavor. In the fermentation process *S. cerevisiae* produces flavor substances such as alcohols and organic acids, which reduce the pH in FCM and improve the palatability. Meanwhile, a large amount of bacteriophage protein will be synthesized by *S. cerevisiae*, and the content of protein in FCM will be increased.

*Bacillus subtilis* with the ability to secrete considerable amounts of enzymes, including proteases, lipase, amylase, and carboxypeptidase is one of the most commonly used bacteria in cottonseed meal fermentation (10). During fermentation, *B. subtilis* can degrade the macromolecular proteins into small molecular peptides and free amino acids, and the content of cellulose and hemicellulose in cottonseed meal could also be degraded. The pH in FCM will be decreased with the production of acidic substances through the metabolism of *B. subtilis*, which inhibited the growth of harmful bacteria and improved palatability. Meanwhile It has a better degradation rate of free gossypol to fermented cottonseed meal with *B. subtilis* (13). Luo et al. used *S. cerevisiae* to ferment cottonseed meal to improve the acid soluble protein content in FCM, up to 16%. Then *B. subtilis* strain1 was used to co-ferment cottonseed meal with *S. cerevisiae*, the ASP contents was improved significantly, up to more than 21% (10, 33). *B. subtilis* M-15 obtained in this study was used in CM fermentation to reduce free gossypol to 51.26 mg/kg, with the free gossypol degradation rate of 93.46%. The crude protein content of FCM was higher than 45.03% and ASP contents was accounted for 29.45% of crude protein. Content of crude fiber, NDF, ADF and ash in FCM decreased significantly and the pH value was reduced to 4.68. This means that fermentation with *B. subtilis* M-15 can improve the quality of cottonseed meal by degrading macromolecular protein, gossypol, crude fiber, NDF, ADF, ash and inhibiting the growth of harmful bacteria.

### Effect of fermentation method on the quality of fermented cottonseed meal

Different fermentation methods have different effects on the quality of FCM. Aerobic solid-state fermentation (ASSF) is mostly used for cottonseed meal fermentation (34). The fermentation time is generally 48–72 h, the fermentation temperature is generally about 30°C, and the degradation rate of free gossypol is about 50% (13). Zhao et al. (2) used *B. subtilis* gj00141 and *S. cerevisiae* gj00079 to degrade more than 59% of free gossypol in cottonseed meal by solid-state fermentation at 30? for 60 h.

The two-stage (aerobic fermentation for 2–3 days, then anaerobic fermentation for 10–12 days) SSF process, which has unique advantages in water and energy savings, decreased weight loss of material, and environmental protection, had not been reported in fermentation of cottonseed meal (35). In this experiment, the anaerobic SSF with *B. subtilis* was used in CM fermentation and attempted to improve CM quality.

Although the duration of anaerobic fermentation is in general much longer than that of the ASSF, the quality of FCM is very similar in the gossypol degradation and acid soluble protein improvement. And anaerobic fermentation even has advantages in this respect. It will be the future direction of the fermentation industry (36, 37). *B. subtilis* BJ-1 and *Rhodococcus rubrum* was used to ferment cotton seed meal with ASSF method, the detoxification rate was 61.91 and 72.54%, respectively, and the content of crude fiber decreased significantly (38). In two stage fermentation mode, which was firstly inoculated with *S. cerevisiae* SC17-1 and *L. plantarum* LP15-1 for anaerobic fermentation and then inoculated *B. subtilis* BS15-3 for aerobic fermentation, the degradation rate of gossypol was 66.28% and the content of acid soluble protein was 4.51% (23). In this experiment, cottonseed meal was fermented by anaerobic SSF with *B. subtilis* M-15. After fermentation, the free gossypol decreased by 93.46% and ASP contents was 13.26%, accounted for 29.45% of crude protein content. Acid soluble proteins (ASP) is lower molecular weight protein hydrolysate, including peptides and free amino acids. After ingested by animals, macromolecular protein is broken down into amino acids and small peptides which are the main form of absorption and utilization by the small intestine. Compared with macromolecular proteins, acid soluble proteins have the characteristics of low energy absorption, fast absorption rate and not easily saturated, which can be directly and safely absorbed in the animal gut system (39).

Several studies have reported the correlation between the nutritional quality of protein feeds such as acid soluble protein and the amount of anti-nutritional factors and their apparent digestibility, concluding that protein feeds with higher crude protein content, acid soluble protein content and few anti-nutritional factors have better apparent digestibility. Ying (40) verified that the *in vitro* digestibility of fermented soybean meal with higher crude protein content, acid soluble protein content and lower anti-nutritional factors contents was better than that of soybean meal. So, it can be inferred that with the improvement of nutritional quality, fermented cotton meal may be beneficial for the digestion of animals.

### Bacterial communities changed during fermentation

In this experiment, high-throughput sequencing of microbial communities in FCM were analyzed to explore changes as a result of inoculation with M-15. After inoculating M-15, the abundance and diversity of bacterial communities decreased. At the phylum level, Firmicutes and Proteobacteria dominated in FCM, especially the proportion of Proteus increased significantly. Proteus and Firmicum are common intestinal bacteria. Firmicutes can produce abundant digestive enzymes, involve in a variety of carbohydrate metabolism, most of them can produce spores, making them resistant to dehydration and extreme environments. Proteobacteria, many members of which produce proteases, can play a role in the cycling of carbon and nitrogen in anaerobic environment (41, 42). At the genus level, the abundance of *Enterobacter* and *Bacillus* increased significantly, to 53.64 and 27.73%, becoming the dominant genus. *Enterobacter* is an important member of Proteobacteria, one of the main intestinal bacteria, both respiratory metabolism and fermentation metabolism. It can participate in the metabolism of many carbon sources such as sugars, organic acids or polyols. *Bacillus*, belonging to Firmicutes, is the main species after 24 h of natural fermentation under aerobic conditions. A large amount of oxygen will be consumed during *Bacillus* sp. growth to create an anaerobic environment and promote the growth of anaerobic species (43, 44). The result of Beta Diversity analysis in this study showed that the difference of the bacterial communities in the three FCM groups is more scattered than that in the CK group, and the flora changes greatly, indicating that the bacterial community in the three FCM groups changes significantly. In a word, the addition of M-15 strains decreased the abundance and diversity of bacteria and increased proportion of the common healthy gut flora in FCM, significantly changed the bacterial community in the FCM groups.

## Conclusion

In this study, *B. subtilis* M-15 with high activity for digesting free gossypol and high protease-producing was screened to degrade free gossypol and improve the nutritional quality of unsterilized CM using anaerobic SSF. The ASP contents increased, while free gossypol contents decreased noticeably after fermentation. The diversity and abundance of the microbial community was reduced and the community structure was simpler significantly. *Enterobacter* sp. and *Bacillus* sp. was the dominant bacterium in FCM. As the results, the strain M-15 can substantially reduce the free gossypol content and improve the nutritional quality of CM through anaerobic solid-state fermentation. In order to evaluate cottonseed meal, the future work is to evaluate the effect of fermented cottonseed meal on animals by measuring digestibility, absorption rate, etc.

## Data availability statement

The datasets presented in this study can be found in online repositories. The names of the repository/repositories and accession number(s) can be found at: https://www.ncbi.nlm.nih.gov/nuccore/HQ401271.1/.

## Author contributions

BZ and JL conceived and designed the experiments. TG determined the physicochemical parameters. ZH analyzed the data and wrote the original draft. JL and XG edited and approved the final manuscript. All authors contributed to the article and approved the submitted version.

## Footnotes

1
http://www.ncbi.nlm.nih.gov/

2
http://drive5.com/uparse/

3
http://www.arb-silva.de

4
http://qiime.org/install/index.html
